# MiR-182-5p protects inner ear hair cells from cisplatin-induced apoptosis by inhibiting FOXO3a

**DOI:** 10.1038/cddis.2016.246

**Published:** 2016-09-08

**Authors:** Yimeng Li, Ao Li, Jingfang Wu, Yingzi He, Huiqian Yu, Renjie Chai, Huawei Li

**Affiliations:** 1 Department of Otorhinolaryngology, Affiliated Eye and ENT Hospital of Fudan University, Shanghai 200031, China; 2 Key Laboratory for Hearing Science, Ministry of Health, Affiliated Eye and ENT Hospital of Fudan University, Shanghai 200031, China; 3 Central Laboratory, Affiliated Eye and ENT Hospital of Fudan University, Shanghai 200031, China; 4 Key Laboratory for Developmental Genes and Human Disease, Ministry of Education, Institute of Life Sciences, Southeast University, Nanjing 210096, China; 5 Co-innovation Center of Neuroregeneration, Nantong University, Nantong 226001, China; 6 Institutes of Biomedical Sciences, Fudan University, Shanghai 200032, China; 7 State Key Laboratory of Medical Neurobiology, Fudan University, Shanghai, China

## Abstract

Cisplatin is widely used for chemotherapy of a variety of malignancies. However, the clinical application of cisplatin is hampered by the resultant irreversible hearing loss due to hair cell apoptosis. To date, no practical regimen to resolve this has been developed. Meanwhile, the role of microRNA in protecting hair cells from cisplatin-induced apoptosis in the inner ear has not been extensively investigated. In this study, we monitored miR-183, -96, and -182 turnover in the cochlea during cisplatin treatment *in vitro*. We found that overexpression of miR-182, but not miR-183 and -96, improved hair cell survival after 3 *μ*M cisplatin treatment *in vitro*. We demonstrated that overexpression of miR-182 repressed the intrinsic apoptotic pathway by inhibiting the translation of FOXO3a. Our study offers a new therapeutic target for alleviating cisplatin-induced hair cell apoptosis in a rapid and tissue-specific manner.

As the cornerstone of platinum-based chemotherapy regimens, cisplatin (cis-diamminedichloroplatinum-II; DDP) is widely used as the first-line treatment of a variety of malignancies, including lung and ovarian cancers and lymphoma.^1, 2^ The application of cisplatin significantly improves patient survival rates, whether as adjuvant treatment combined with surgery or salvage treatment for unresectable disease.^3, 4^ However, the use of cisplatin is severely limited by its unwanted side effects, including ototoxicity and nephrotoxicity, which reduce patient tolerance during treatment and interfere with the long-term quality of life.^5, 6^ The cytotoxicity of cisplatin is mediated by induction of apoptosis.^7, 8^

In the inner ear, cisplatin leads to irreversible hair cell loss, together with damage to both the spiral ganglion and stria vascularis.^6, 9, 10^ The mechanism of cisplatin-induced apoptosis is unclear.^2^ The most frequently used strategy to relieve cisplatin-induced ototoxicity is the use of antioxidants and anti-inflammatory agents.^11, 12^ However, the treatments mentioned above have remained at the pre-clinical stage due to their limited curative effects or at the undesirable expense of tumoricidal efficacy. Therefore, further understanding of cisplatin-induced apoptosis is necessary for the development of new strategies to protect hair cells.

MicroRNAs (miRNAs) are a class of endogenous non-coding RNAs 20–22 nucleotides in length. They negatively regulate gene expression at the post-transcriptional level by binding to partially complementary sites in the 3′-untranslated regions (3′-UTR) of target messenger RNAs (mRNAs). miRNAs have important roles in regulating many biological processes, such as proliferation, differentiation, and cell death.^13, 14^ In vertebrates, the expression of the highly conserved miR-183/96/182 cluster (hereafter, miR-183/96/182) is predominant in ciliated sensory organs.^15, 16^ In the inner ear, their expression is confined to hair cells and the spiral ganglion.^17, 18^ Previous studies have revealed the importance of miR-183-5p, -96-5p, and -182-5p (hereafter, miR-183, -96, and -182) in hair cell development and maintenance.^19, 20^ Other studies conducted in cancer cells have reported their anti-apoptotic effects.^21, 22^ However, the roles of miR-183, -96, and -182 in protecting hair cells from cisplatin-induced apoptosis have not been investigated.

In this study, we monitored the kinetic changes in miR-183, -96, and -182 levels during cisplatin treatment in organ of Corti explants. MiR-182 overexpression had a protective effect against cisplatin-induced hair cell apoptosis *in vitro*. Furthermore, miR-182 overexpression inhibited the expression of FOXO3a in hair cell nuclei. We also identified a direct interaction between miR-182 and FOXO3a by immunoprecipitation assay. Therefore, miR-182 might protect inner ear hair cells from cisplatin-induced apoptosis through inhibition of FOXO3a.

## Results

### Rapid turnover of miR-183, -96, and -182 under cisplatin treatment

**Figure 1:**
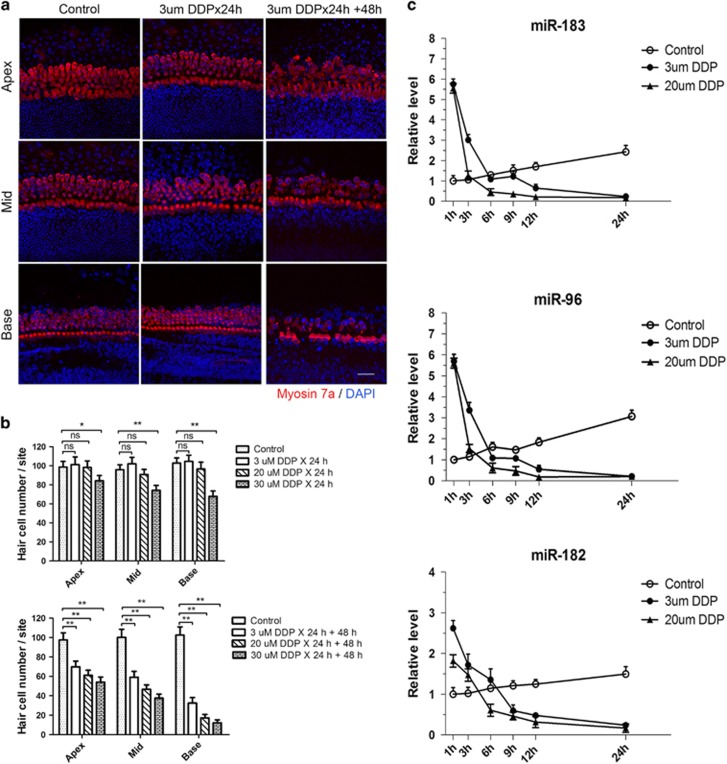
Turnover of miR-183, -96, and -182 levels during cisplatin treatment. (**a**, **b**) Hair cell number did not change significantly after 24 h of treatment with 3 and 20 *μ*M cisplatin. Significant hair cell loss was observed at 48 h after cisplatin washout following treatment with 3, 20, and 30 *μ*M cisplatin. (**c**) MiR-183, -96, and -182 levels increased significantly after 1 h of cisplatin treatment and decreased markedly in the following 23 h. The miRNA level of the control group at 1 h was set as 1. Data are shown as means±S.E. One-way ANOVA followed by Newman–Keuls test, **P<0.01, *P<0.05. Scale bar in bottom-right corner: 20 *μ*m

First, we investigated the extent of hair cell loss according to cisplatin concentration. Organ of Corti explants were cultured in medium containing 3, 20, or 30 *μ*M cisplatin for 24 h. Hair cells were counted at 0, 24, and 48 h after cisplatin treatment. No significant hair cell loss was observed in the 3 and 20 *μ*M groups at 0 h after cisplatin treatment. However, when cochleae were cultured for a further 48 h after cisplatin washout, significant hair cell loss was observed at all cisplatin concentrations (Figures 1a and b).

To investigate the roles of miR-183, -96, and -182 in cisplatin-induced hair cell apoptosis, we monitored miR-183, -96, and -182 expression levels during 24 h of 3 and 20 *μ*M cisplatin treatment using quantitative reverse-transcription PCR (qRT-PCR). Interestingly, although the number of hair cells remained similar, we observed considerable turnover of miR-183, -96, and -182 levels during the first 24 h of cisplatin treatment. The miR-183, -96, and -182 levels increased rapidly and peaked at 1 h, then decreased rapidly during the following 5 h. The miR-183/96/182 expression level decreased to lower than that of the cisplatin-untreated groups at 6 h, and continued to decrease from 6 to 24 h. Moreover, the rate of decrease in miR-183/96/182 expression was positively related to the concentration of cisplatin (Figure 1c). The miR-183, -96, and -182 levels increased spontaneously in the control group during culture for 24 h (Figure 1c).

### Overexpression of miR-182 prevented cisplatin-induced hair cell loss *in vitro*

**Figure 2:**
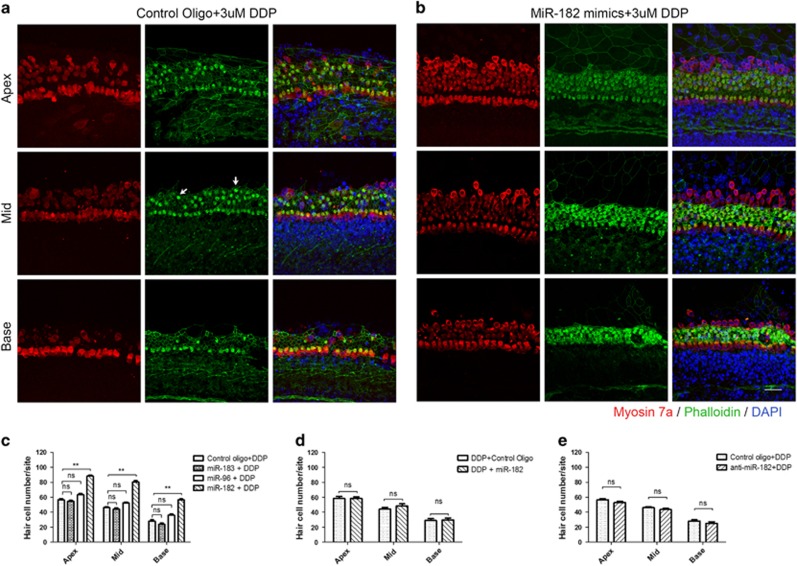
Overexpression of miR-182 before treatment with 3 *μ*M cisplatin significantly improved hair cell survival. (**a**–**c)** Surviving hairs were confirmed by phalloidin and myosin 7a double staining. The stereocilia of many hair cells had been lost and the cuticular plates had shrunk (white arrowhead) after cisplatin treatment (**a**, **b**) Residual hair cell numbers at the apex, mid, and base turn in the miR-183 OE, miR-96 OE, miR-182 OE, and control groups 48 h after cisplatin washout (**c**). (**d)** Residual hair cell numbers in the miR-182 rescue and control groups. (**e**) Residual hair cell numbers in the miR-182 knockdown and control groups 48 h after cisplatin washout. Data are shown as means±S.E. Student's *t*-test and one-way ANOVA followed by Newman–Keuls test, **P<0.01. Scale bar in bottom-right corner: 20 *μ*m. ns, no significance

The marked turnover of miR-183/96/182 suggested their importance in hair cell death in the presence of cisplatin. We hypothesized that the elevation of miR-183, -96, and -182 levels during the first hour is a self-defense response to the cytotoxicity of cisplatin and may protect hair cells. To test this hypothesis, miR-183, -96, and -182 were overexpressed in cochlear explants by transfecting miRNA mimics using lipid transfection reagent before treatment with 3 or 20 *μ*M cisplatin. qRT-PCR was used to determine the transfection efficiency of miRNA mimics. After transfection with miR-183, -96, and -182 mimics, robust increases in miRNA levels were observed at days 1, 3, and 5 compared with the control group (Supplementary Figures 1A–C), which indicated that the ectopically introduced miRNA mimics were maintained at a high level for at least 5 days. After transfection, cochlear explants were cultured in serum-free medium for 24 h before treatment with 3 or 20 *μ*M cisplatin for 24 h. Surviving hair cells were counted 48 h after cisplatin washout. Interestingly, protective effects were evident only in the miR-182 overexpression group following treatment with 3 *μ*M cisplatin but not with 20 *μ*M cisplatin (Figures 2a–c). To determine whether overexpression of miR-182 could rescue hair cell death after cisplatin exposure, cochlear explants were transfected with miR-182 mimics after 24 h of treatment with 3 *μ*M cisplatin. No significant difference was observed in the miR-182 rescue group compared with the control group (Figure 2d).

Finally, to determine the effect of miR-182 knockdown on hair cell survival under cisplatin treatment, anti-miR-182 mimics were transfected to cochlear explants to knockdown endogenous miR-182. qRT-PCR showed that the miR-182 level was significantly inhibited at days 1, 3, and 5 after anti-miR-182 transfection (Supplementary Figure 1d). Hair cell counts showed no significant difference between the miR-182 knockdown group and the control group transfected with control oligos at 48 h after cisplatin washout (Figure 2e).

### Overexpression of miR-182 inhibited cisplatin-induced mitochondrial-dependent apoptosis of hair cells *in vitro*

**Figure 3:**
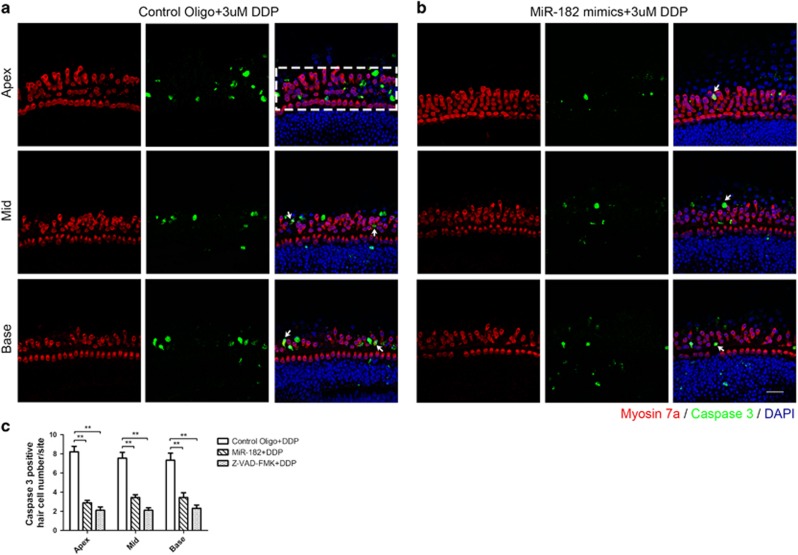
Overexpression of miR-182 significantly reduced cisplatin-induced hair cell apoptosis. (**a**–**c**) Caspase 3 (+) cells in the white-dashed-line box are considered caspase 3 (+) hair cells. Caspase 3 (+) and Myo 7a (+) double-labeled cells (hair cells undergoing apoptosis) are indicated by white arrowheads (**a**, **b**). Numbers of apoptotic hair cells at the apex, mid, and base turn in the control group, miR-182 OE group, and z-VAD-fmk group (**c**). Data are shown as means±S.E. Student's *t*-test, **P<0.01. Scale bar in bottom-right corner: 20 *μ*m

To assess cisplatin-induced hair cell apoptosis, we used immunofluorescence staining of cleaved caspase 3, a common executor in the apoptotic pathway.^23^ Cochlear explants were transfected with miR-182 mimics or control oligos, and then treated with 3 *μ*M cisplatin for 24 h. Apoptotic hair cells were quantified 24 h after cisplatin washout. In addition, z-Val-Ala-Asp-fluoromethylketone (z-VAD-fmk), a broad-spectrum caspase inhibitor, which was shown to inhibit hair cell apoptosis in a mouse model in which hair cells undergo apoptotic self-degeneration,^24^ was used to determine the involvement of caspase-dependent apoptosis of hair cells under cisplatin treatment. Significantly fewer caspase 3-positive hair cells were observed in the miR-182 overexpression group (2.95±0.83 for apex (*n*=27, *P*<0.01), 3.45±0.93 for mid (*n*=27, *P*<0.01), and 3.47±1.54 for base (*n*=27, *P*<0.01)) and in the z-VAD-fmk group (2.11±1.05 for apex (*n*=27, *P*<0.01), 2.26±0.78 for mid (*n*=27, *P*<0.01), and 2.34±1.02 for base (*n*=27, *P*<0.01)) compared with the control group (8.24±1.72 for apex (*n*=27), 7.65±1.85 for mid (*n*=27), and 7.37±2.28 for base (*n*=27); Figures 3a–c).

**Figure 4:**
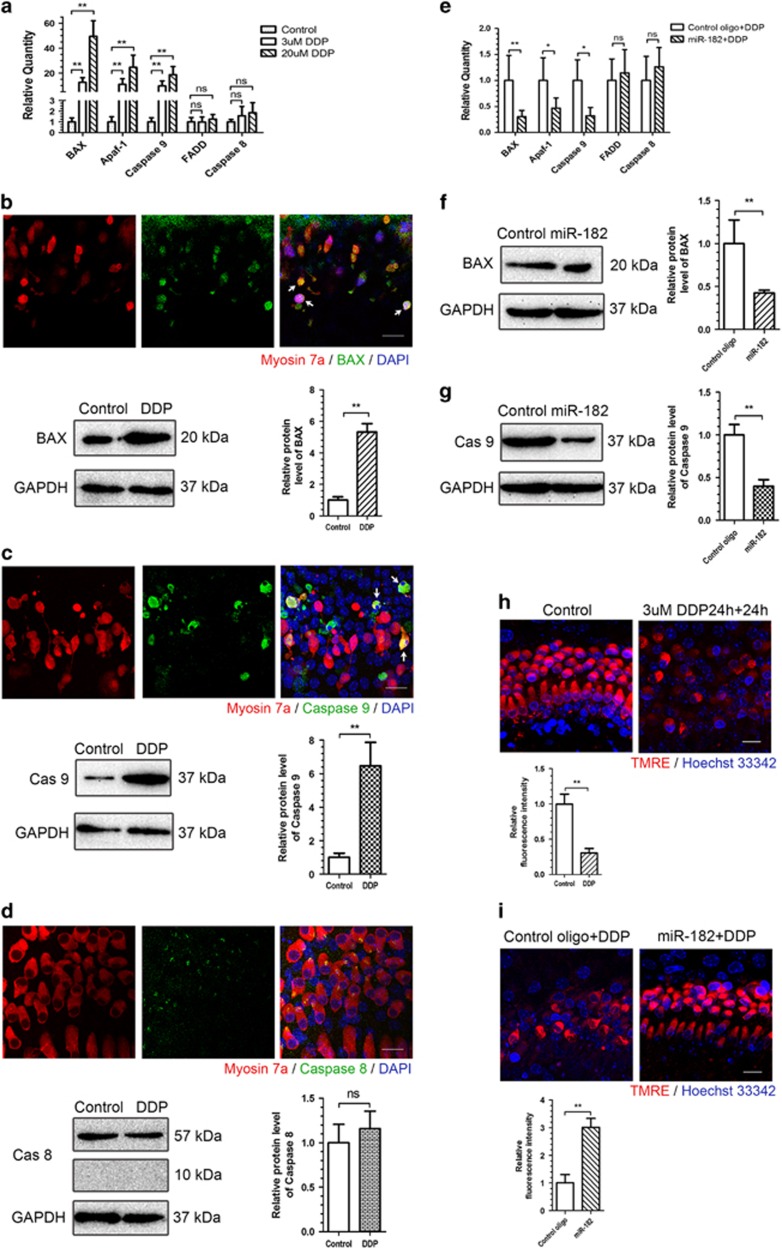
Overexpression of miR-182 inhibited cisplatin-induced mitochondrial-dependent apoptosis of hair cell. (**a**) Bax, Apaf-1, and caspase 9 levels were significantly increased at 24 h of cisplatin washout, whereas FADD and caspase 8 levels were not significantly different. (**b**, **c)** At 24 h of cisplatin washout, clear induction of Bax and caspase 9 was exhibited in hair cells (white arrows indicate the double staining of Bax and myosin 7a (**b**, upper panel) or caspase 9 and myosin 7a (**c**, upper panel)). Western blot shows the increased protein levels of Bax (**b**, lower panel) and caspase 9 (**c**, lower panel) after cisplatin treatment. (**d**) No significant of caspase 8 expression in hair cells or increased caspase 8 protein level after cisplatin treatment. (**e**) The mRNA levels of Bax, Apaf-1, and caspase 9 (**e**) and the protein levels of Bax and caspase 9 (**f**, **g**) were significantly reduced in the miR-182 overexpression group at 24 h after cisplatin washout, whereas FADD and caspase 8 mRNA levels were not significantly different (**e**). (**h**) Significant reduction of TMRE staining in hair cells treated with cisplatin. (**i**) The fluorescence intensity of TMRE staining in miR-182 overexpression group is significantly higher than control group. Data are shown as means±S.E. Student's *t*-test and one-way ANOVA followed by Newman–Keuls test, **P<0.01, *P<0.05. Scale bar in bottom-right corner: 10 *μ*m (**b**, **c**, **d**). Scale bar in bottom-right corner: 20 *μ*m (**h**, **i**). ns, no significance

The mitochondrial-dependent intrinsic pathway and the death-receptor-dependent extrinsic pathway are the two major apoptotic pathways.^25^ To investigate the effects of cisplatin treatment on these two apoptotic pathways in the inner ear, qRT-PCR, immunofluorescence staining, and western blotting were performed to assess the levels of several key apoptotic effectors, including BCL2-associated X protein (Bax), apoptotic peptidase activating factor 1 (Apaf-1), and caspase 9 of the intrinsic pathway, as well as FAS-associated protein with a death domain (FADD) and caspase 8 of the extrinsic pathway. At 24 h after cisplatin washout, relative Bax, Apaf-1, and caspase 9 mRNA (Figure 4a) and protein (Figures 4b and c) levels increased significantly, whereas those of FADD and caspase 8 were not significantly different from the control group (Figures 4a and d).

To determine the effects of miR-182 overexpression on the intrinsic and extrinsic apoptotic pathways after cisplatin treatment, qRT-PCR and western blotting were performed. The increases in Bax, Apaf-1, and caspase 9 expression were significantly inhibited in the miR-182 overexpression group, whereas FADD and caspase 8 expression were unaffected (Figures 4e–g).

Mitochondria have a decisive role in the intrinsic apoptotic pathway. The mitochondrial transmembrane potential, which reflects mitochondrial function, was assessed by tetramethylrhodamine ethyl ester (TMRE) staining assay.^26^ The fluorescence intensity in the cisplatin-treated group was 0.31±0.07-fold than that of the control group (*P*<0.01; Figure 4h). However, when transfected with miR-182 mimics before cisplatin treatment, the fluorescence intensity was 2.98±0.31-fold higher than that of the control group transfected with control oligo (*P*<0.01; Figure 4i).

### Overexpression of miR-182 did not alter the function of mechanotransduction channels in hair cells

**Figure 5:**
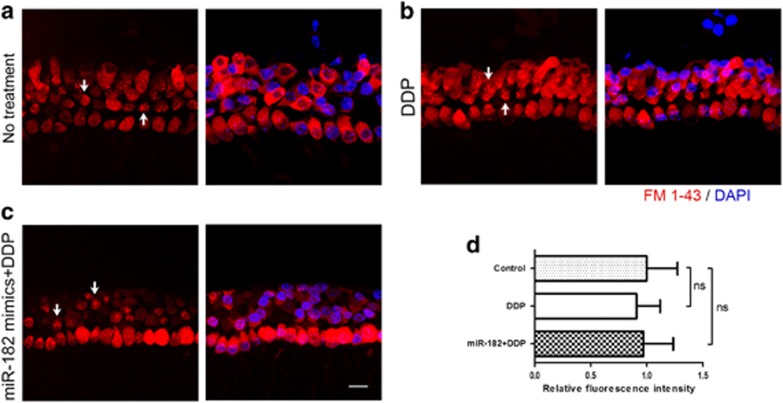
Overexpression of miR-182 did not alter the function of the mechanotransduction channels of hair cells. (**a**–**d**) After transfection with miR-182 mimics followed by treatment with cisplatin for 24 h (**c**), hair cells took up FM 1-43 to a similar level as the control group (**a**) and the cisplatin-only group (**b**). Bright fluorescent punctae (white arrowheads) show the locations of mechanotransduction channels. Scale bar in bottom-right corner: 20 *μ*m. ns, no significance

Functional mechanotransduction is required for cisplatin-induced hair cell death in the zebrafish lateral line.^27^ To determine whether miR-182 protects hair cells from cisplatin-induced damage by reducing cisplatin uptake through blocking of mechanotransduction channels, we performed an FM 1-43 staining assay. Hair cells in the miR-182 overexpression group took up the FM 1-43 dye, as well as normal hair cells and those in the cisplatin-only group. In addition, bright fluorescent punctae were seen at the top of hair cells where the mechanotransduction channels are located (Figures 5a–d), which indicated that they were functional.

### MiR-182 protected hair cells against cisplatin-induced apoptosis through inhibition of FOXO3a

**Figure 6:**
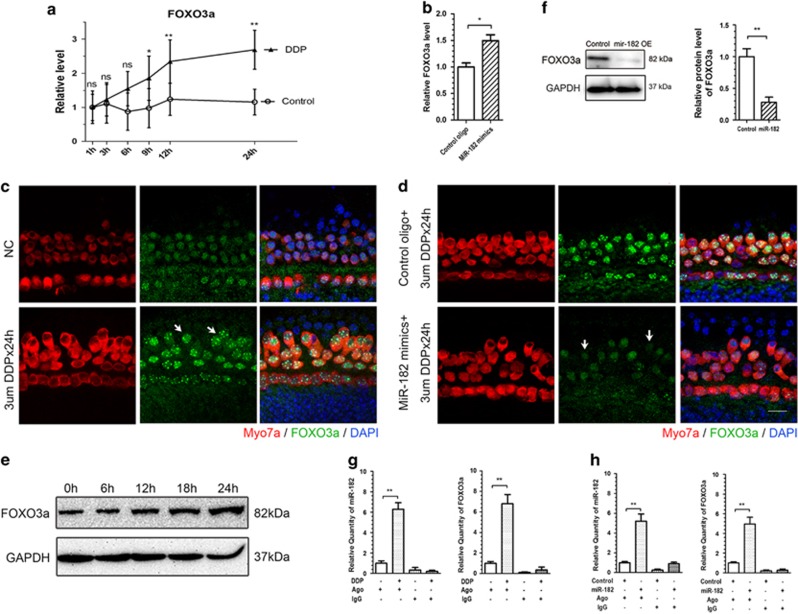
MiR-182 protects hair cells from cisplatin-induced apoptosis through inhibition of FOXO3a. (**a**) The FOXO3a mRNA levels increased gradually during the 24 h of cisplatin treatment. (**b)** Transfection with miR-182 mimics before treatment with cisplatin for 24 h did not inhibit the increase in FOXO3a mRNA level. (**c**) FOXO3a expression is significantly upregulated in hair cell nuclei. Bright fluorescence indicates chromosome (white arrowhead). (**d**) FOXO3a expression is significantly lower in the miR-182 OE group (white arrowhead) compared with the control group. (**e**) Western blot analysis showed that the FOXO3a protein level were 1.02±0.34, 0.85±0.52, 1.87±0.64 (*P*<0.01, *n*=3), 3.66±1.23 (*P*<0.01, *n*=3), and 5.25±1.38-fold (*P*<0.01, *n*=3) at the 0, 6, 12, 18, and 24 h, respectively, of cisplatin treatment compared with the control group. (**f**) After transfection with miR-182 mimics followed by treatment with cisplatin for 24 h, the FOXO3a protein level of the miR-182 OE group was 0.28±0.08-fold (*P*<0.01, *n*=3) that of the control group. (**g**) The relative level of miR-182 and FOXO3a mRNA co-precipitated with Ago2 protein was 6.28±0.64-fold (*P*<0.01, *n*=3) and 6.8±0.89-fold than that of the control group, respectively, after 24 h of cisplatin treatment. (**h**) After transfection with miR-182 mimics followed by treatment with cisplatin for 24 h, the level of miR-182 and FOXO3a mRNA co-precipitated with Ago2 was 5.2±0.72-fold (*P*<0.01, *n*=3) and 4.97±0.67-fold (*P*<0.01, *n*=3) than that of the control group, respectively. Data are shown as means±S.E. Student's *t*-test, **P<0.01, *P<0.05. Scale bar in bottom-right corner: 10 *μ*m. ns, no significance

miRNAs implement their regulatory roles in animals and plants by post-transcriptional repression of their target genes.^13^ To screen for the underlying target gene of miR-182, we searched the miRanda-mirSVR and TargetScan 6.1 databases for proposed target genes of miR-182 that are involved in regulating apoptosis. We also retrieved previous reports of target genes of miR-182. Both FOXO3a (ref. 28) and breast cancer type 1 susceptibility protein (BRCA1)^29^ are validated targets of miR-182 and are related to apoptosis (Supplementary Figure 2). We performed qPCR to investigate the effect of cisplatin treatment on FOXO3a and BRCA1 expression in cochlear explants. FOXO1, a member of the FOXO protein superfamily reported to be regulated by miR-183 and -96,^30, 31^ was also included. The mRNA levels of FOXO3a (Figure 6a) and FOXO1 (Supplementary Figure 3A) were significantly increased during 24 h of cisplatin treatment, but the expression level of BRCA1 was unaffected (Supplementary Figure 3B). Then, we performed immunofluorescent staining and western blotting to assess FOXO3a and FOXO1 protein levels in a spatial and quantitative manner. As a transcription factor, FOXO3a exerts its function by translocation into the nucleus.^32^ Before cisplatin treatment, the nuclei and cytoplasm of hair cells showed immunofluorescent staining for FOXO3a (Figure 6c, upper panel). After 24 h of cisplatin treatment, the fluorescence in hair cell nuclei increased markedly (Figure 6c, lower panel). However, no distinct fluorescent staining of FOXO1 in hair cell nuclei was observed before or after cisplatin treatment (Supplementary Figure 3D). Western blotting showed that the FOXO3a protein level increased gradually and reached 5.25±1.38-fold (*n*=3, *P*<0.01) higher than that of the control group after 24 h of cisplatin treatment. No significant increase in FOXO1 protein level was observed after cisplatin treatment (Supplementary Figure 3F).

Next, we explored the effects of miR-182 overexpression on cisplatin-induced FOXO3a upregulation in hair cells. Overexpression of miR-182 did not inhibit the increase in FOXO3a (Figure 6b) and FOXO1 (Supplementary Figure 3C) mRNA levels induced by cisplatin treatment. However, immunofluorescent staining and western blotting showed significant inhibition of FOXO3a protein in hair cell nuclei in the miR-182 overexpression group compared with the control group (Figures 6d and f). Overexpression of miR-182 did not alter FOXO1 expression in hair cell nuclei (Supplementary Figure 3E and G).

miRNAs induce post-transcriptional regulation of target genes by forming RNA-induced silencing complexes (RISCs) with Argonaute 2 (Ago2) protein.^33, 34^ To elucidate the direct interaction of miR-182 and FOXO3a, RNA-binding protein immunoprecipitation (RIP) analyses were performed using rabbit anti-Ago2 antibody to co-precipitate miRNA and its target mRNAs bound to RISC. After treatment with cisplatin for 1 h, the relative miR-182 and FOXO3a mRNA levels co-precipitated with Ago2 protein were 6.28±1.15-fold (*n*=3, *P*<0.01) and 6.8±1.54-fold (*n*=3, *P*<0.01), respectively, greater than that in the undamaged control group (Figure 6g), confirming the interaction of miR-182 and FOXO3a during cisplatin treatment. Next, we explored whether the downregulation of FOXO3a in the miR-182 overexpression group was due to a direct interaction of miR-182 and FOXO3a. After transfection with miR-182 mimics and treatment with cisplatin for 1 h, the relative levels of miR-182 and FOXO3a mRNA co-precipitated with Ago2 protein were 5.2±1.25-fold (*n*=3, *P*<0.01) and 4.97±1.16-fold (*n*=3, *P*<0.01), respectively, greater than the control group (Figure 6h), confirming direct repression of FOXO3a by miR-182.

### MiR-182 attenuated the synergetic effect of PI3K inhibitor and cisplatin on hair cell loss

**Figure 7:**
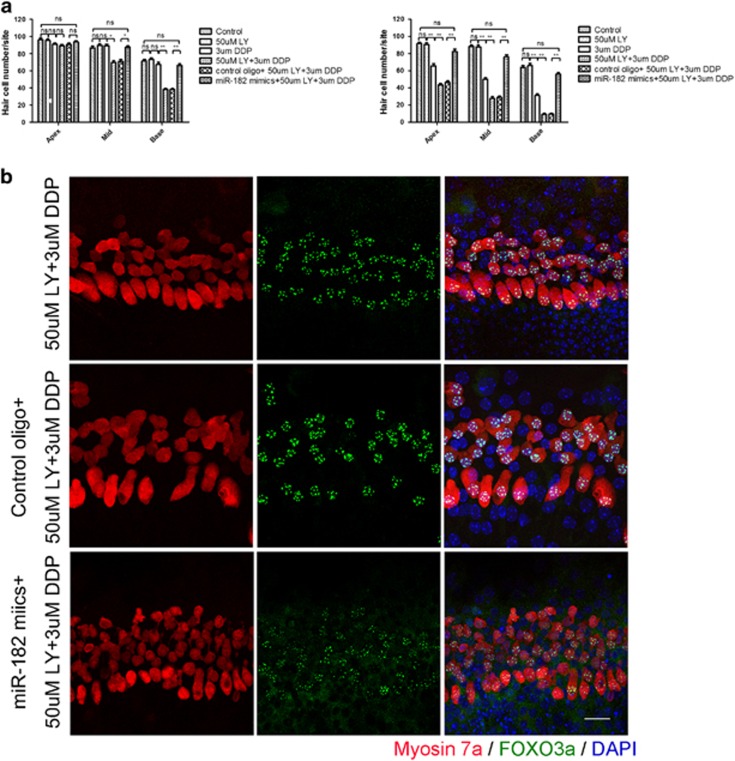
MiR-182 attenuated the synergetic effect of PI3K inhibitor and cisplatin on hair cell loss. (**a**) The synergetic effect of PI3K inhibitor and cisplatin on hair cell loss and the protective effect of miR-182. Loss of hair cells was significantly exacerbated when organ of Corti explants were treated with LY294002 and cisplatin in combination at 24 h. Transfection with miR-182 mimics before LY294002 and cisplatin treatment significantly improved hair cell survival. (**b**) Intense expression of FOXO3a in the hair cell nuclei in cochlear explants treated with cisplatin and LY294002 together (**b**, upper panel) and control oligo group (**b**, middle panel), whereas lower fluorescence intensity in hair cell nuclei was observed in miR-182 overexpression group (**b**, lower panel). Data are shown as means±S.E. One-way ANOVA followed by Newman–Keuls test, **P<0.01. Scale bar in bottom-right corner: 20 *μ*M. ns, no significance

The PI3K–AKT pathway is recognized as a main regulator of the FOXO protein family. AKT inhibits the function of FOXO as a transcription factor by preventing translocation to nuclei and binding to promoter regions through phosphorylation of FOXO protein.^35, 36^ The PI3K inhibitor LY294002 aggravates gentamicin-induced hair cell loss,^37^ but the exact mechanism has not been elucidated. To determine the effect of PI3K inhibitor on cisplatin-induced hair cell loss, cochlear explants were treated with cisplatin alone or in combination with LY294002, and similar aggravation of hair cell loss was observed in the cisplatin and LY294002 combination group compared with the cisplatin-alone group (Figure 7a). In addition, clear FOXO3a expression was detected in the hair cell nuclei by immunofluorescence staining when treated with LY294002 and cisplatin together (Figure 7b). Next, we transfected cochlear explants with miR-182 mimics before LY294002 and cisplatin treatment to determine the effect of miR-182 overexpression on hair cell loss. Interestingly, overexpression of miR-182 significantly improved hair cell survival in the presence of both LY294002 and cisplatin (Figure 7a). Lower FOXO3a fluorescence staining in hair cell nuclei was observed in the miR-182 overexpression group compared with the control group transfected with control oligo (Figure 7b, lower panel).

## Discussion

Cisplatin is one of the most widely used antitumor drugs. However, its poor selectivity toward tumor cells results in serious side effects, such as nephrotoxicity and ototoxicity. There is at present no effective medical treatment for ototoxicity.^38^ Present trials are at the pre-clinical stage, mainly because of concerns regarding interference with the antitumor effect of cisplatin. Thus, novel protective agents with higher selectivity toward non-cancerous cells are required.

miRNAs have important roles in regulating cell death, particularly apoptosis. Efforts have been made to improve cell survival by modulating miRNA levels, which yielded promising results.^39, 40, 41^ In the inner ear, a recent study identified miR-207, which directly targets Akt3, as a pro-apoptotic miRNA in an ionizing radiation-induced hair cell death model.^42^ Moreover, miR-34a/SIRT1/p53 signaling is activated while that of the miR-183 family is downregulated during age-related hair cell apoptosis.^43, 44^ Thus, protecting hair cells against apoptosis by downregulating pro-apoptotic miRNAs or upregulating anti-apoptotic miRNAs is a feasible strategy.

Previous studies indicated important roles for the miR-183, -96, and -182 cluster in inner ear development and pathogenesis.^45, 46, 47^ We observed rapid turnover of miR-183, -96, and -182 levels during the first 24 h of cisplatin treatment. The majority of studies have reported that the miR-183 family exerts a protective effect against apoptosis; however, others have reported that miR-183, -96, and -182 facilitate apoptosis.^48, 49, 50^ We hypothesized that the rapid elevation of miR-183, -96, and -182 levels during the first hour of cisplatin treatment was due to the self-defense response of hair cells to cisplatin. Activation of the apoptotic cascade is decided by the balance between the pro-apoptotic and anti-apoptotic factors within and around cells. If cisplatin-induced pro-apoptotic effects overwhelm the intrinsic defensive mechanism of hair cells, irreversible apoptosis will result. Thus, miR-183, -96, and -182 levels decreased markedly between 2 and 24 h after cisplatin treatment, leading to hair cell apoptosis. Moreover, miR-183, -96, and -182 levels increased spontaneously in the control group. This was likely due to self-adaptation of the cochlear explants upon transfer from *in vivo* to *in vitro*, which may facilitate hair cell survival during the change in growth environment.

Assessment of the effects of miRNA overexpression partially validated our hypothesis of a protective effect of miR-182 against cisplatin-induced apoptosis of hair cells. Overexpression of miR-182 before cisplatin treatment significantly improved hair cell survival; however, overexpression of miR-183 and -96 failed to increase the hair cell survival rate. Although miR-183, -96, and -182 are located in a genomic DNA fragment of <4 kb and co-transcribed from genomic DNA, they do not target the same group of genes.^51^ miRNAs regulate their targets by partial complementary binding to the 3′-UTR) of mRNAs with their ‘seed' sequence,^52^ and the ‘seed' sequences of miR-183, -96 and, -182 are different, so their targets are different. Moreover, one miRNA could target a group of genes, so we conjecture that the miR-183, -96, and -182 cluster is co-transcribed to maintain the balance of a larger group of genes. Thus, their functions are diverse and complementary rather than overlapping. In addition, the protective effect of miR-182 was observed only in organ of Corti explants treated with a low concentration (3 *μ*M) of cisplatin, not a high concentration (20 *μ*M). This reflects the notion that miRNAs regulate their target genes in a ‘mild' pattern.^53, 54^

We observed significant increases in the expression of Bax, Apaf-1, and caspase 9, three key effectors of the intrinsic apoptotic pathway, after 24 h of cisplatin treatment, whereas the levels of FADD and caspase 8, dominant members of the death-receptor-dependent extrinsic pathway, were not significantly changed. Bax is a pro-apoptotic factor that initiates the intrinsic apoptotic pathway by permeabilizing the mitochondrial outer membrane.^55^ Apaf-1 is another important factor responsible for activating caspase 9 in the intrinsic apoptotic pathway.^56^ Loss of mitochondrial transmembrane potential was observed using a TMRE staining assay when organ of Corti explants were treated with cisplatin. These findings are consistent with previous reports that the intrinsic mitochondrial-dependent pathway is the dominant pathway in cisplatin-induced hair cell apoptosis.^57^ Furthermore, overexpression of miR-182 before cisplatin treatment significantly inhibited the increase of Bax, Apaf-1, and caspase 9, but not of FADD and caspase 8, expression, as well as the increased fluorescence intensity in the TMRE staining assay, indicating that miR-182 protects hair cells from cisplatin-induced apoptosis by inhibiting the intrinsic apoptotic pathway.

Uptake of cisplatin is the initial and pivotal event in its cytotoxicity.^2^ Cisplatin enters hair cells through a variety of routes, including copper transporters, organic cation transporters, and mechanotransduction channels.^58, 59^ In our study, surviving hair cells maintained their ability to take up FM 1-43, suggesting that the protective effect of miR-182 on hair cells is not mediated by blocking mechanotransduction channels.

Immunofluorescence staining and western blotting yielded spatial and quantitative evidence that FOXO3a participates in cisplatin-induced hair cell apoptosis. FOXO3a is a pro-apoptotic transcription factor that functions by activating BIM, a pro-apoptotic member of the BCL-2 protein family.^60, 61^ BIM activates Bax to induce mitochondrial-dependent apoptosis.^62^ We observed marked elevation of FOXO3a and Bax expression during the initial phase of cisplatin-induced hair cell apoptosis. Overexpression of miR-182 reduced the expression of FOXO3a in hair cell nuclei. Moreover, the co-precipitation of miR-182 and FOXO3a mRNA by RIP assay indicated the direct regulation of FOXO3a by miR-182 in cisplatin-induced hair cell apoptosis. Thus, we believe that miR-182 suppresses hair cell apoptosis through direct inhibition of FOXO3a.

Finally, the finding that overexpression of miR-182 attenuates the synergetic effect of a PI3K inhibitor and cisplatin on hair cell loss revealed the underlying mechanism. PI3K inhibitor enhances cisplatin-induced hair cell apoptosis by reducing the phosphorylation of FOXO3a by PI3K–AKT. Overexpression of miR-182 repressed the expression of FOXO3a, a pro-apoptotic transcription factor and direct target of the PI3K–AKT signaling pathway, which alleviated hair cell death.

In summary, we found that among the miR-183/96/182 family, overexpression of only miR-182 enhanced hair cell survival during treatment with a low concentration (3 *μ*M) of cisplatin *in vitro*. The protective effect was achieved by inhibiting intrinsic mitochondrial-dependent apoptosis via repression of FOXO3a. Our findings indicate the possibility of protecting hair cells before cisplatin therapy using endogenous, tissue-specific miRNAs. These findings may provide new therapeutic targets for the prevention of cisplatin-induced hair cell death, and may ameliorate the retardant effect of protective agents against the antitumor effect of cisplatin. However, the precise *in vivo* effect and whether miRNA treatment would be effective against the therapeutic dose of cisplatin warrant further studies.

## Materials and Methods

### Animals

Postnatal day 0–3 BALB/c mice of both sexes were obtained from the Department of Laboratory Animal Science of Fudan University. The care and use of animals were in strict accordance with the ‘Guiding Directive for Humane treatment of Laboratory Animals' issued by the Chinese National Ministry of Science and Technology. The Institutional Animal Care and Use Committee of Fudan University approved all procedures. All possible efforts were made to minimize the number of animals used and their suffering.

### Organ of Corti explants

Cochlear cultures were obtained as described previously.^63, 64^ Briefly, mice were killed by decapitation. The organs of Corti were dissected from cochleae in ice-cold phosphate-buffered saline (PBS; HyClone, Logan, UT, USA) and placed on glass coverslips pre-coated with poly-D-lysine (Sigma-Aldrich, St Louis, MO, USA). Cultures were maintained in DMEM/F-12 medium (Gibco, Carlsbad, CA, USA) supplemented with N2 and B27 (both from Invitrogen, Carlsbad, CA, USA) at 37 °C in a 5% CO_2_ atmosphere. Fetal bovine serum (5% Invitrogen) was added to the medium for only the first 24 h of culture to facilitate cell attachment.

### Cisplatin treatment, miRNA mimic transfection, z-VAD-fmk, and LY294002 treatment

All drugs and oligonucleotides were diluted in serum-free culture medium. Three concentrations of cisplatin (Sigma; 3, 20, and 30 *μ*M) were used in this study. Cochlear explants were cultured in cisplatin-containing medium for 24 h and washed three times for 5 min each in PBS following completion of cisplatin treatment.

miRNA mimics and inhibitors were synthesized by GenePharma Co, Ltd. (Shanghai, China). The sequences were as follows: miR-183 (5′-UAUGGCACUGGUAGAAUUCACU-3′); miR-96 (5′-UUUGGCACUAGCACAUUUUUGCU-3′); miR-182 (5′-UUUGGCAAUGGUAGAACUCACACCG-3′); anti-miR-182 (5′-CGGUGUGAGUUCUACCAUUGCCAAA-3′); and control (5′-UUGUACUACACAAAAGUAGUC-3′). Oligonucleotides were transfected to organ of Corti explants using the K2 transfection system (Biontex, München/Laim, Germany) following the manufacturer's instructions at 80 nM for miRNA mimics and 100 nM for miRNA inhibitors. The cochlear explants were transfected with miRNA mimics or inhibitors for 24 h. After washing in PBS three times for 5 min each, cochlear explants were cultured in serum-free medium for a further 24 h and then treated with cisplatin.

The cochlear explants were incubated with z-VAD-fmk (100 *μ*M; Apexbio, Boston, MA, USA) for entire culture duration.

LY294002 (50 *μ*M; Selleck Chemicals, Houston, TX, USA) was applied to cochlear explants alone or together with 3 *μ*M cisplatin for 24 h as reported previously^37^ to assess their effects on hair cell loss.

### Immunofluorescence

Cochlear explants were fixed in 4% paraformaldehyde for 10 min then washed thoroughly in PBS. After permeabilization in 0.5% Triton X-100 in PBS for 30 min and blocking in 10% donkey serum in PBS for 1 h at room temperature, the explants were incubated with the primary antibody overnight at 4 °C. The primary antibodies used in this study were as follows: rabbit polyclonal anti-myosin 7a (1:500) (Proteus Biosciences, Ramona, CA, USA); mouse monoclonal anti-myosin 7a (1:500) (Developmental Studies Hybridoma Bank, Iowa City, IA, USA); rabbit polyclonal anti-cleaved caspase 3 (1:400) (Cell Signaling Technology Inc., Beverly, MA, USA); rabbit monoclonal anti-Bax (1:400) (Cell Signaling Technology); rabbit monoclonal anti-caspase 9 (1:500) (Cell Signaling Technology); rabbit monoclonal anti-caspase 8 (1:200) (Cell Signaling Technology); rabbit monoclonal anti-FOXO1 (1:200) (Cell Signaling Technology); and rabbit monoclonal anti-FOXO3a (1:500) (Cell Signaling Technology). Primary antibodies were decanted in PBS and detected using TRITC- or Alexa Fluor 488-conjugated secondary antibodies (1:800) (Jackson ImmunoResearch, West Grove, PA, USA). Culture explants were stained with Alexa Fluor 488-conjugated phalloidin (1:800) (Invitrogen) for 40 min to label the hair bundles and cuticular plates of hair cells. Cell nuclei were counterstained with DAPI (Invitrogen). All fluorescence images were obtained using a Leica TCS SP8 confocal laser-scanning microscope (Leica, Heidelberg, Germany).

### Enumeration of surviving and apoptotic hair cells

Myosin 7a- and Alexa Fluor 488-conjugated phalloidin staining were used for quantitative analysis of surviving hair cells after cisplatin treatment. Caspase 3 staining was used to label apoptotic cells. Caspase 3-positive cells located within the four rows of hair cells and on the same plane as myosin-7a-positive hair cells were counted as hair cells undergoing apoptosis. The numbers of surviving and apoptotic hair cells in each × 800 microscopic field (~150 *μ*m along the long axis of the cochlear duct) were counted. Three microscopic fields were randomly selected from the apical, middle, and basal turns in each cochlear explant. At least nine cochleae from each experimental group from three independent experiments were evaluated.

### Quantitative reverse-transcription PCR

Six cochlear cultures from each experimental group were pooled for RNA extraction. Total RNA was extracted using TRIzol reagent (Invitrogen) following the manufacturer's instructions. The quantity and quality of RNA were assessed using a Nanodrop 2000 spectrophotometer (Thermo Scientific, Waltham, MA, USA) and 0.8% agarose gel electrophoresis. qRT-PCR of miRNAs was performed using the polyA-tailing method as reported previously.^65, 66^ One microgram of total RNA was tailed using *Escherichia coli* polyA polymerase (New England Biolabs, Ipswich, MA, USA). cDNA was synthesized using the GoScript Reverse Transcription System (Promega, Madison, WI, USA). Reverse transcription (RT) of miRNAs was conducted using an anchor RT primer. mRNAs were reverse-transcribed using the RT primer provided in the RT kit. qPCR was performed using GoTaq qPCR Master Mix (Promega) on an ABI 7500 Real-Time PCR system (Applied Biosystems, Foster City, CA, USA). All primers used in this study are listed in Table 1. Small nuclear RNA U6 and glyceraldehyde-3-phosphate dehydrogenase (GAPDH) were used as internal controls for miRNA and mRNA quantification, respectively. Relative quantities were calculated using the 2-ΔΔCT method.^67^

### TMRE staining assay

Cochlear explants were removed from the culture medium and briefly washed in PBS, followed by incubation in TMRE (100 nM in culture medium; Invitrogen) for 20 min at 37 °C in a 5% CO_2_ atmosphere. Samples were rinsed twice with PBS. Cell nuclei were stained with Hoechst 33342 for 10 min. TMRE staining was visualized in living cochlear explants using a confocal laser microscope.

### FM 1-43 uptake

Cochlear explants were removed from the culture medium and briefly washed in PBS, then incubated in FM 1-43FX (5 *μ*g/ml in ice-cold PBS; Invitrogen) for 30 s. Samples were rinsed twice with PBS and fixed. After counterstaining with DAPI, FM 1-43 staining was visualized using a confocal laser microscope.

### Western blotting analysis

Fifteen cochlear explants of each experimental group were lysed using an ultrasonic vibrator in 150 *μ*l ice-cold Western Lysis Buffer (Beyotime, Nantong, China) for total protein extraction. Approximately 30 *μ*g of total protein were fractionated by 8% SDS-PAGE and transferred to a PVDF membrane (Millipore, Billerica, MA, USA). Target protein was probed with primary antibodies overnight at 4 °C. The primary antibodies were as follows: rabbit monoclonal anti-Bax (1:1000) (Cell Signaling Technology); rabbit monoclonal anti-caspase 9 (1:1000) (Cell Signaling Technology); rabbit monoclonal anti-caspase 8 (1:1000) (Cell Signaling Technology); rabbit anti-FOXO1 (1:1000) (Cell Signaling Technology); rabbit anti-FOXO3a (1:1000) (Cell Signaling Technology); and rabbit anti-GAPDH (1:5000) (Cell Signaling Technology). HRP-conjugated anti-rabbit IgG was used as the secondary antibody (1:5000) (Cell Signaling Technology). Bands were visualized using the ECL kit according to the manufacturer's instructions (Pierce, Thermo Scientific).

### Ago2 immunoprecipitation

Fifteen explants from each experimental group were lysed using an ultrasonic vibrator in 500 *μ*l ice-cold RIP lysis buffer (300 mM NaCl, 5 mM MgCl_2_, 0.1% NP-40 and 50 mM Tris HCl, pH 7.5, 1 mM dithiothreitol, 1 mM PMSF, 100 units/ml RNasin (Promega), and protease inhibitor cocktail (Roche, Basel, Switzerland) on ice). The lysate was centrifuged at 14 000 × *g* at 4 °C for 10 min. A volume of 10 *μ*l rabbit monoclonal anti-Ago2 antibody (Cell Signaling Technology) or 10 *μ*g normal rabbit IgG (Santa Cruz Biotechnology, Dallas, TX, USA) were added to the supernatant and gently rotated overnight at 4 °C. Protein A Beads slurry (50 *μ*l; Santa Cruz Biotechnology) was added and incubated for 3 h at 4 °C with gentle rotation. Beads were pelleted at 2500 r.p.m. for 5 min, the supernatant was removed, and beads were washed three times in 1 ml ice-cold wash buffer (150 mM NaCl, 1 mM MgCl_2_, 0.05% NP-40, and 50 mM Tris HCl; (pH 7.5)) for 10 min each. The wash buffer was decanted, and bound material was eluted from beads with 50 *μ*l of 0.1 M glycine (pH 2.3) for 10 min at room temperature. Eluted fractions were neutralized immediately with an equal volume of 1 M Tris-HCl (pH 8) and then treated with 20 U of proteinase K for 10 min at 55 °C. Co-precipitated RNA was extracted using TRIzol reagent (Invitrogen). RNA (100 ng) was then subjected to miRNA and mRNA quantification.

### Statistical analysis

All experiments were performed independently in triplicate. All data are shown as means±S.E.M. Student's *t*-test and one-way ANOVA with a Newman–Keuls test were used to determine the significance of differences between two experimental groups or differences among more than two groups. Values of *P*<0.05 were considered to indicate statistical significance.

## Figures and Tables

**Table 1:** Primers used in reverse transcription and qPCR for miRNAs and mRNAs

*Name*	*Sequence*
Anchor RT primer	5′-GCTGTCAACGATACGCTACCTAACGGCATGACAGTG(T)15V-3′
Universal resverse primer	5′-GCTGTCAACGATACGCTACCTA-3′
U6 forward	5′-CGCTTCGGCAGCACATATACTAA-3′
MiR-183 forward	5′-GTATGGCACTGCTAGAATTCACT-3′
MiR-96 forward	5′-TTTGGCACTAGCACATTTTTGC-3′
MiR-182 forward	5′-TGGCAATGGTAGAACTCACACC-3′
Bax forward	5′-TGAAGACAGGGGCCTTTTTG-3′
Bax resverse	5′-AATTCGCCGGAGACACTCG-3′
Apaf-1 forward	5′-AGTGGCAAGGACACAGATGG-3′
Apaf-1 resverse	5′-GGCTTCCGCAGCTAACACA-3′
Caspase 9 forward	5′-TCCTGGTACATCGAGACCTTG-3′
Caspase 9 reverse	5′-AAGTCCCTTTCGCAGAAACAG-3′
FADD forward	5′-CTGCGCCGACACGATCTAC-3′
FADD reverse	5′-CGGGCCAGTCTTTTCCAGT-3′
Caspase 8 forward	5′-TGCTTGGACTACATCCCACAC-3′
Caspase 8 resverse	5′-TGCAGTCTAGGAAGTTGACCA-3′
FOXO1 foward	5′-ATGCTCAATCCAGAGGGAGG-3′
FOXO1 reverse	5′-ACTCGCAGGCCACTTAGAAAA-3′
FOXO3 forward	5′-CTGGGGGAACCTGTCCTATG-3′
FOXO3 reverse	5′-TCATTCTGAACGCGCATGAAG-3′
BRCA1 forward	5′-CTGCCGTCCAAATTCAAGAAGT-3′
BRCA1 reverse	5′-CTTGTGCTTCCCTGTAGGCT-3′
GAPDH forward	5′-TGGCCTTCCGTGTTCCTAC-3′
GAPDH reverse	5′-GAGTTGCTGTTGAAGTCGCA-3′

## Abbreviations

DDP: cisplatin, cis-diamminedichloroplatinum-II
3′-UTR: 3′-untranslated regions
FOXO: forkhead box transcription factor
z-VAD-fmk: z-Val-Ala-Asp-fluoromethylketone
Bax: BCL2-associated X protein
Apaf-1: apoptotic peptidase activating factor 1
FADD: FAS-associated protein with a death domain
TMRE: tetramethylrhodamine ethyl ester
BRCA1: breast cancer type 1 susceptibility protein
RISCs: RNA-induced silencing complexes
Ago2: Argonaute 2
RIP: RNA-binding protein immunoprecipitation
