# Supplementary material for: MiR-182-5p protects inner ear hair cells from cisplatin-induced apoptosis by inhibiting FOXO3a
Source: Cell Death Dis. 2016 Sep 8;7(9):e2362–. doi: 10.1038/cddis.2016.246 (PMC5059852; doi:10.1038/cddis.2016.246)
Supplement: Supplementary Figures [file cddis2016246x1.pdf]

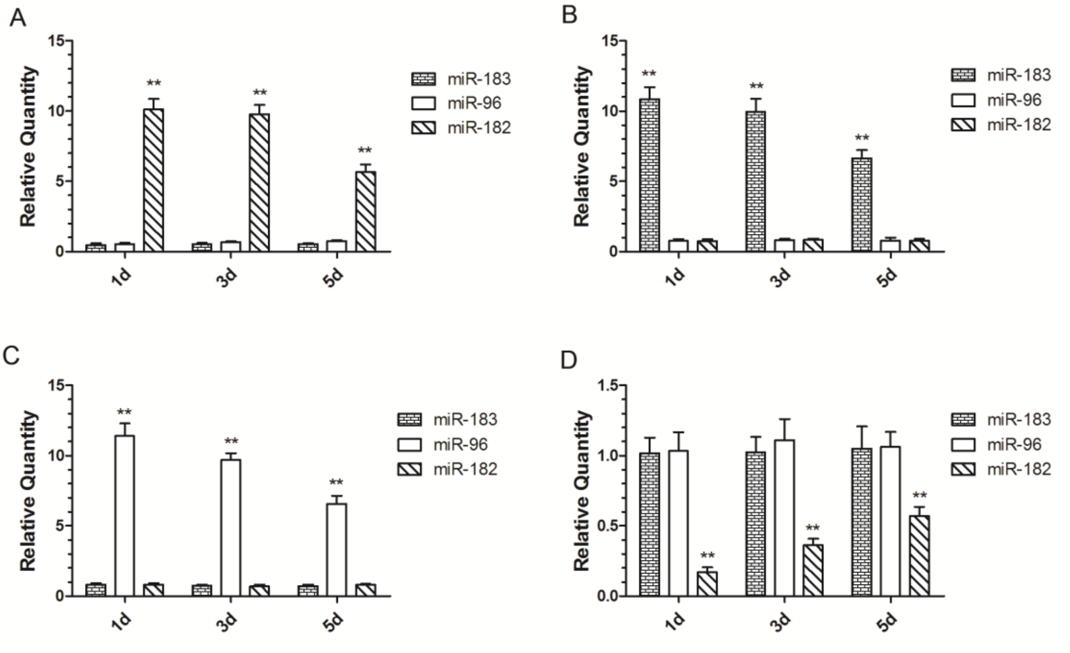

Supplemental Figure 1: A–C. miR-183, miR-96 and miR-182 levels were significantly increased for 5 days after transfection with miR-182, miR-183 and miR-96 mimics. **D.** The miR-182 level was significantly reduced at 5 days after transfection with anti-miR-182. Data are shown as means ± S.E. Student's *t*-test, ** = P<0.01.

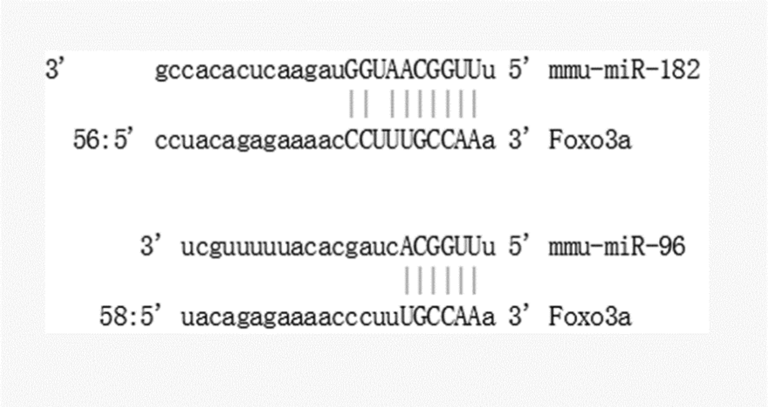

Supplemental Figure 2: The *in silico*-predicted binding site of miR-182 and miR-96 in the 3'-UTR of FOXO3a.

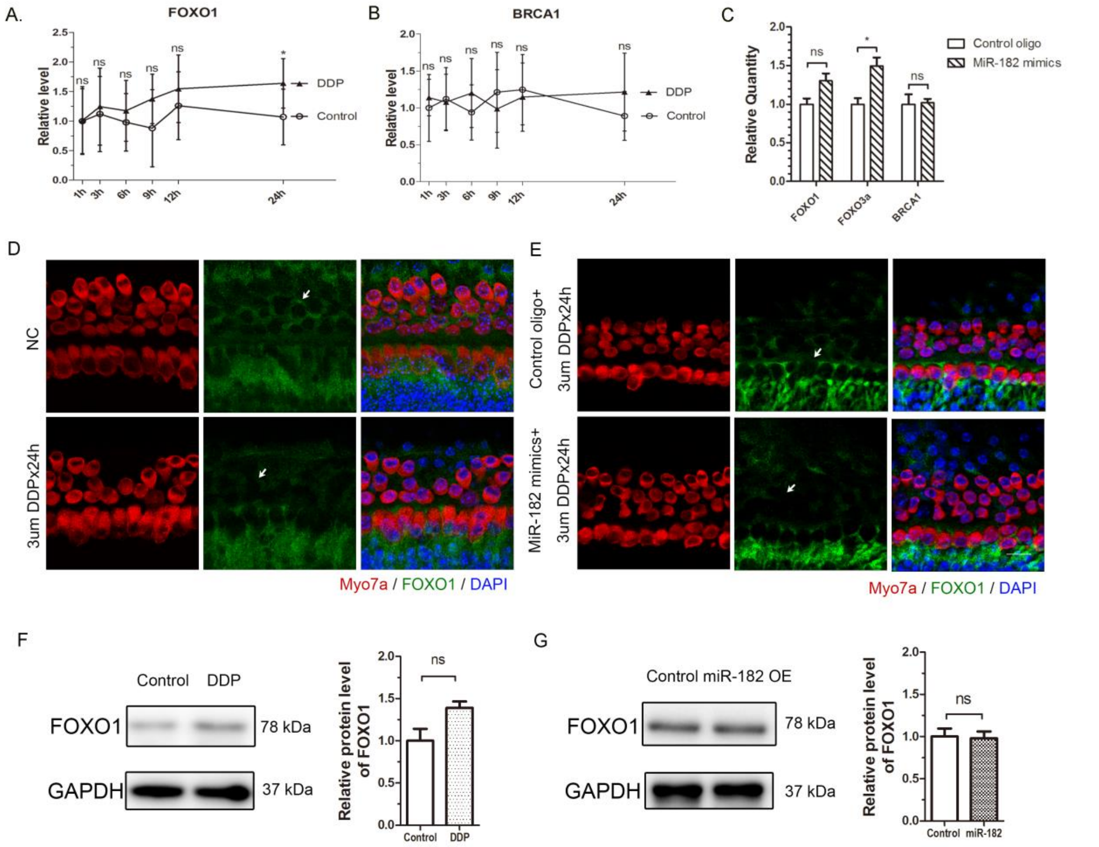

Supplemental Figure 3: FOXO1 and BRCA 1 are not involved in the response of hair cells to cisplatin cytotoxicity. **A.** Slight increase of FOXO1 mRNA level during the 24h of cisplatin treatment. **B.** No significant differences in BRCA 1 mRNA level during the 24h of cisplatin treatment. C. Transfection with miR-182 mimics before treatment with cisplatin for 24 h did not inhibit the increase in FOXO1 and BRCA 1 mRNA level. **D, E** No significant FOXO1 immunofluorescent staining (white arrowheads) was observed in hair cell nuclei irrespective of whether they were treated with cisplatin alone or transfected with miR-182 mimics or control oligos. **F.** After treatment with cisplatin for 24 h, Western blot analysis showed no significant difference in the FOXO1 protein level compared to the control group. **G.** After transfection with miR-182 mimics followed by treatment with cisplatin for 24 h, there

was no significant difference in the FOXO1 protein level between the miR-182 OE group and control group. Data are shown as means ± S.E. Student's *t*-test.
